# Bridging the Gap in Understanding Bone Metastasis: A Multifaceted Perspective

**DOI:** 10.3390/ijms25052846

**Published:** 2024-02-29

**Authors:** Basant Elaasser, Nour Arakil, Khalid S. Mohammad

**Affiliations:** Department of Anatomy, College of Medicine, Alfaisal University, Riyadh 1153, Saudi Arabia; belaasser@alfaisal.edu (B.E.); narakil@alfaisal.edu (N.A.)

**Keywords:** bone metastases, bone microenvironment, bone remodeling, metabolic reprogramming, therapy

## Abstract

The treatment of patients with advanced cancer poses clinical problems due to the complications that arise as the disease progresses. Bone metastases are a common problem that cancer patients may face, and currently, there are no effective drugs to treat these individuals. Prostate, breast, and lung cancers often spread to the bone, causing significant and disabling health conditions. The bone is a highly active and dynamic tissue and is considered a favorable environment for the growth of cancer. The role of osteoblasts and osteoclasts in the process of bone remodeling and the way in which their interactions change during the progression of metastasis is critical to understanding the pathophysiology of this disease. These interactions create a self-perpetuating loop that stimulates the growth of metastatic cells in the bone. The metabolic reprogramming of both cancer cells and cells in the bone microenvironment has serious implications for the development and progression of metastasis. Insight into the process of bone remodeling and the systemic elements that regulate this process, as well as the cellular changes that occur during the progression of bone metastases, is critical to the discovery of a cure for this disease. It is crucial to explore different therapeutic options that focus specifically on malignancy in the bone microenvironment in order to effectively treat this disease. This review will focus on the bone remodeling process and the effects of metabolic disorders as well as systemic factors like hormones and cytokines on the development of bone metastases. We will also examine the various therapeutic alternatives available today and the upcoming advances in novel treatments.

## 1. Introduction

Bone metastasis plays a crucial role in the advancement of cancer, indicating a shift to a more aggressive and sometimes untreatable phase of the illness. However, we still have a limited understanding of the complex process through which cancer spreads to the skeletal system and how metastatic cancer cells interact with the specific bone microenvironment [1,2,3,4]. Bone remodeling processes, which are interrupted by the invasion of cancer cells, give rise to pathological bone lesions. Based on their specific effects on bone tissue, these lesions can be categorized into osteolytic and osteoblastic types. This classification reflects the outcome of the interaction between cancer cells and bone remodeling.

It is essential to analyze the molecular pathways and signaling networks that enable cancer cells to migrate and establish themselves in the bone marrow environment. A key aspect in understanding the process of bone metastasis is the recognition of the significant impact of the ‘vicious cycle’. This cycle involves the reciprocal interactions between tumor cells and bone-residing cells, such as osteoblasts and osteoclasts, resulting in both bone degradation and tumor development [5,6,7]. This review focuses on analyzing the impact of both local and systemic variables, such as cytokines and growth factors, as well as the metabolic reprogramming of cancer cells and bones on the advancement of bone metastases.

The clinical ramifications of bone metastasis are extensive, including difficulties in timely identification, diagnosis, and therapy. We discuss the current methods used for diagnosing medical conditions, including imaging and biomarker analysis. We also examine the newest breakthroughs in targeted treatments and medications that affect bone structure. The substantial influence of bone metastases on the quality of life of patients is also investigated, focusing on the treatment of pain and the avoidance of skeletal-related events.

This review aims to provide a comprehensive comprehension of bone metastasis by using knowledge derived from contemporary research in order to improve clinical care and therapeutic approaches. We aim to cultivate a thorough comprehension of this intricate illness, thus facilitating future advancements in the provision of care and therapy for people suffering from bone metastases.

## 2. Bone Remodeling

### 2.1. Definition and Purpose

Bone remodeling is an essential physiological process that is crucial for preserving the structural integrity of bone, balancing systemic mineral concentrations, and accommodating increased mechanical stresses and demands on the bone [8]. Two seemingly opposing yet complementary processes tightly regulate the cycle of bone remodeling: the removal of old or damaged bone and its replacement with a new bone. Various factors, both systemic and local, rigorously control the rate of bone resorption and bone formation, to ensure no net change in bone mass. A significant shift in the balance between formation and resorption leads to bone mass loss or gain. This might manifest with fractures, bone pain, or compressive symptoms. Bone remodeling is a dynamic, lifelong process that continuously occurs, albeit with fluctuations in remodeling rates across different stages of life, hormonal changes, physical activity, and even across different bones in the body.

### 2.2. The Main Players of Bone Remodeling

The bone remodeling process is complex, and despite numerous new discoveries, its mechanisms remain to be fully elucidated [9]. Bone remodeling involves three primary phases: resorption, reversal, and formation. These steps represent the overarching stages of the remodeling cycle, describing the general sequence of events. Each of these phases involves multiple steps with intricate cellular and molecular events. Despite being thought of as having distinct stages, bone remodeling is an integrated process occurring both continuously and simultaneously. 

Although bone looks like an inert structure, it features a metabolically active, dynamic, and changing microscopic environment. The bone matrix comprises a mix of organic and inorganic components making a rigid yet tensile bone structure. Collagen, mainly type I, constitutes most of the organic component of the bone, with inorganic minerals, mostly calcium and phosphate, embedded within the collagen matrix [10,11]. 

The main cells at play in bone remodeling are osteoblasts, osteoclasts, osteocytes, and lining cells [12]. Osteoclasts are large, multinucleated cells derived from hematopoietic stem cells. Macrophage colony-stimulating factor (M-CSF) and the receptor activator of nuclear factor kappa beta ligand (RANKL) are necessary for the commitment to the osteoclast lineage [13]. Mononuclear osteoclast precursors express receptor activator of nuclear factor-kappa B (RANK), a receptor that signals through the NFκB pathway. The binding of RANK to RANKL activates the receptor. RANKL is an essential requirement for osteoclastogenesis [14,15]. A study suggested that RANKL expressed by osteocytes is more important in supporting osteoclastogenesis than that expressed by osteoblasts [16]. Osteoclasts have a pivotal role in the breaking down of dysfunctional or old bone and releasing calcium, phosphate, and growth factors from the bone matrix. Osteoblasts, in contrast to osteoclasts, are responsible for the formation and mineralization of new bone. However, they can direct bone resorption through the activation of osteoclasts. Osteoblasts are derived from mesenchymal stem cells. Mesenchymal stem cells display considerable plasticity, enabling them to differentiate into cells of different lineages, such as osteoblasts, chondrocytes, adipocytes, and myocytes, through different transcription factors [13]. The commitment of mesenchymal stem cells to differentiate into osteoblasts depends on runt-related transcription factor 2 (RUNX2), β-catenin, and osterix [17,18]. Osteocytes are terminally differentiated osteoblasts embedded within the bone matrix. They are the most abundant long-living bone cells, comprising 90% to 95% of all bone cells. In the absence of bone remodeling, osteocytes exert an inhibitory effect on both osteoblasts and osteoclasts. Osteocytes inhibit osteoclastogenesis by secreting sclerostin and transforming growth factor-beta (TGFβ). Sclerostin, a protein encoded by the *SOST* gene, functions as an inhibitor of canonical Wnt signaling pathways, leading to the inhibition of bone formation [19,20,21]. 

### 2.3. The Process of Bone Remodeling

Bone remodeling can be categorized into either targeted or stochastic (nontargeted) remodeling [22]. Stochastic remodeling, mediated by systemic factors such as parathyroid hormone (PTH), thyroxine, and estrogen, does not induce remodeling in a specific location. It is believed to play a role in serum calcium homeostasis. Local factors mediated targeted remodeling to induce bone remodeling on a specific site. The bone remodeling process can be divided into five major phases: activation, resorption, reversal, formation, and termination.

#### 2.3.1. Activation Phase

The remodeling process is initially triggered by a remodeling signal, which can either be a systemic factor (such as hormones) or a local factor (such as bone microdamage from increased mechanical strain) [23]. Osteocytes, stellate-shaped cells residing within bone lacunae, have dendrite-like processes extending within the canaliculi, forming an extensive lacuna–canalicular network. This network enables osteocytes to sense mechanical stimuli and transmit cellular signals to other osteocytes and osteoblasts on the bone surface [24]. This allows osteocytes to regulate osteoblast and osteoclast activity. Osteocyte apoptosis secondary to bone matrix microdamage leads to the recruitment of osteoclast and osteoblast precursors to the area of microdamage [25]. These groups of cells—osteocytes, osteoblasts, osteoclasts, and various other cells—constitute a basic multicellular unit (BMU). A canopy of cells encases the BMU, forming a defined area termed the bone remodeling canopy (BRC) [26,27]. The BRC is nourished by capillaries believed to serve as pathways for circulating osteoblasts and osteoclast precursors, facilitating their migration to regions undergoing active remodeling [28]. The BRC acts as a microenvironment to facilitate the coupling of bone resorption and formation. Bone formation follows bone resorption in equal amounts across both time and place, ensuring no net change in bone mass because of physiological remodeling [29]. When osteoblast-mediated bone formation and osteoclast-mediated bone resorption are uncoupled, bone mass is lost. This might lead to either generalized bone loss or focal osteolysis, as observed in various conditions, such as bone metastases and Paget’s disease of bone. The disinhibition of osteocytes leads to the recruitment of osteoclast precursors and osteoblast activation [30]. Osteocyte apoptosis lowers sclerostin and TGFβ levels, removing the inhibition of osteoclastogenesis [31,32]. The consequent expression and release of RANKL and M-CSF by damaged osteocytes further drive osteoclastogenesis. As previously explained, the recruitment of mononuclear osteoclast precursors from the bone marrow occurs, and they undergo differentiation and activation.

#### 2.3.2. Resorption Phase

Osteoblasts modulate osteoclastogenesis through RANKL/OPG interactions, and immune system elements, inflammatory cytokines (IL-1, TNFα, and IL-6), and interferon-β also impact osteoclast differentiation [33]. While the RANKL pathway is necessary, it is not solely sufficient, and osteoblasts additionally regulate osteoclast differentiation through immunoglobulin-like receptors. Mature osteoclasts adhere to the bone surface, forming an annular sealing zone [34]. Protons are released via vacuolar ATPase, facilitated by carbonic anhydrase II. Matrix metalloproteinases (MMPs) and cathepsin K contribute to the degradation of the mineralized bone matrix, creating resorption pits or Howship’s lacunae. Growth factors, such as TGFβ and insulin groeth factors (IGF), are released from the degraded bone matrix. Following resorption, osteoclasts undergo apoptosis [35,36,37]. 

#### 2.3.3. Reversal Phase

The reversal phase refers to the switch from bone resorption to bone formation [38]. Bone-lining cells on inactive bone surfaces can transform into osteoblasts when mechanically stimulated or influenced by intermittent parathyroid hormone (PTH) [38]. Cells of an osteoblastic lineage, called reversal cells, remove the unmineralized bone matrix left behind by osteoclasts and prepare the surface of the bone for the deposition of a new bone matrix by osteoblasts. Reversal cells display osteoblast cell markers, including RUNX2, alkaline phosphatese (ALP), and collagen 3 (Col3) [39]. Macrophages, including osteomacs, are also likely involved in matrix debris removal during the reversal phase through MMP production and phagocytosis [23]. This phase prepares for new osteoblast-driven bone formation through the deposition of the “cement line,” facilitated by osteopontin [40]. Reversal surfaces exhibit higher cell density than quiescent surfaces, and this enrichment is crucial for coupling and bone formation initiation [38]. The collaboration between osteomacs and mesenchymal bone-lining cells may facilitate events during the reversal phase, with the last role of reversal cells involving signaling for the transition from bone resorption to formation within the BMU [23].

#### 2.3.4. Formation Phase

Bone formation involves the laying down of unmineralized osteoid rich in type I collagen in resorption pits by osteoblasts [41]. Osteoblasts facilitate the mineralization of the osteoid by incorporating hydroxyapatite crystals. While this mechanism is not fully understood, tissue nonspecific alkaline phosphatase and ectonucleotide pyrophosphatase play a role.

#### 2.3.5. Termination Phase

The process of mineralization, also known as calcification, is the last step, and it starts around thirty days after the development of the osteoid. ALP and phosphoprotein kinases are the enzymes that are responsible for regulating the mineralization process. The mineralization of the bone matrix that is not already mineralized is indirectly stimulated by vitamin D [42]. 

During the 90 days that follow osteoid deposition, this process is completed in trabecular bone, but in cortical bone, it is completed 130 days later. The bone enters the quiescent phase after mineralization, and the quantity of bone created is equal to the amount of bone that was resorbed from the environment [43]. Following their function, mature osteoblasts face one of three fates: apoptosis, becoming bone-lining cells covering the periosteum, or being embedded into the bone matrix and terminally differentiating into osteocytes [44].

Several bone remodeling mechanisms are unexplored. Notably, the molecular details of the transition from resorption to formation, including reversal cell regulatory mechanisms, require more study. Systemic aspects in targeted remodeling, especially in pathological circumstances like osteoporosis and arthritis, need further study. Understanding how systemic hormonal signals and local mechanical stress responses affect bone remodeling might provide novel treatment targets.

### 2.4. Systemic Factors Influencing Bone Remodeling

Hormonal influences on bone remodeling are significant. In understanding how hormones impact bone health, several key players influence bone remodeling through specific molecular actions [8,45]. 

Parathyroid hormone (PTH) and PTH-related peptide (PTHrP) contribute to bone remodeling by interacting with the common receptor PTH1R, expressed in bone and cartilage cells [46]. They exert anabolic effects on bone formation, influencing osteoblast proliferation and differentiation and preventing apoptosis through molecular mechanisms such as IGF-1 synthesis and Wnt signaling. 

Calcitonin inhibits osteoclastic bone resorption and may influence osteoblastic bone formation through indirect effects on Wnt10b expression [47]. 

Estrogen regulates bone metabolism by attenuating osteoclastogenesis, stimulating osteoclast apoptosis, and inhibiting osteoblast apoptosis, crucial for maintaining bone mass [48]. Androgens play key roles in bone remodeling, influencing growth plate maturation, as well as trabecular and cortical bone mass, and indirectly inhibiting osteoclast activity through effects on the RANKL/RANK/OPG system [49]. 

Thyroid hormones, including triiodothyronine (T3), impact bone turnover, and their effects are mediated by thyroid hormone nuclear receptors (TRs), particularly TRα and TRβ, influencing osteoblast and osteoclast activities [50]. Glucocorticoids (GCs) accelerate bone resorption and reduce bone formation, acting through GR signaling in preosteoblast/stromal cells and osteoblasts, leading to glucocorticoid-induced osteoporosis (GIO) [8]. Growth hormone (GH) affects bone remodeling by stimulating osteoblast proliferation, collagen production, and bone formation, with direct and indirect effects on bone resorption through IGF-1 or IGFBP. Overall, these hormones play crucial roles in maintaining bone homeostasis through intricate molecular mechanisms.

Cytokines play a pivotal role in bone remodeling by influencing both osteoblasts and osteoclasts. Tumor necrosis factor-alpha (TNFα) and interleukin-1 (IL-1) stimulate M-CSF and MCP-1, attracting osteoclasts, while TNFα also promotes systemic RANKL production [51]. IL-1 induces prostaglandin E2 (PGE2) synthesis and affects RANKL expression, crucial for osteoclast formation. Both cytokines contribute to osteoclast maturation. Interleukin-6 (IL-6) correlates with joint erosion in rheumatoid arthritis, stimulating RANKL production and potentially influencing bone resorption, especially in estrogen-deficient conditions. Macrophage colony-stimulating factor (M-CSF) is essential for osteoclast formation, produced by stromal cells and osteoblasts under various stimuli. Additionally, the LIF/IL-6 cytokine subfamily, including IL-11, has variable effects on bone cells, stimulating osteoclast numbers [52]. IL-17, secreted by Th17 cells, may upregulate osteoclast activity and stimulate mesenchymal stem cells for potential bone regeneration [53]. IFN-γ, identified in rheumatoid arthritis, inhibits osteoclast formation and maturation, influencing osteoblasts and mesenchymal stem cells in a complex manner, promoting osteogenic differentiation but, in synergy with TNFα, inhibiting both osteogenic and adipogenic differentiation. These cytokines, often released in inflammatory conditions, impact bone remodeling by regulating the balance between osteoclast and osteoblast activities, shedding light on potential therapeutic targets for conditions like osteoporosis and bone-related diseases [54]. Particularly, rheumatoid arthritis (RA) affects the bone remodeling process [51]. In RA, synovial macrophages release pro-inflammatory cytokines like TNFα, IL-1, and IL-6, promoting bone resorption. Additionally, RANKL expression in RA synovial fibroblasts contributes to osteoclast differentiation.

Tissue damage triggers an immediate immune response, involving innate immune cells like neutrophils, mast cells, monocytes, and macrophages, followed by adaptive immune cells such as T and B cells [53,55,56]. In bone regeneration, these immune cells play distinct roles. Acute inflammation, for instance, prompts chemokine production, expediting bone formation in fractures. Conversely, chronic inflammation inhibits the factors supporting bone formation, contributing to osteolytic lesions. Neutrophils, abundant in the blood, are pivotal for early bone repair, although their dysregulation can lead to bone-related issues [57]. Macrophages, derived from monocytes, significantly contribute to various phases of bone healing and regeneration. M1 macrophages aid in inflammation, while M2 macrophages support ossification. Dendritic cells, innate lymphoid cells, and T cells, along with B cells, influence bone regeneration through intricate interactions. The dynamics of these immune cells in bone remodeling are complex and contingent upon specific physiological conditions.

### 2.5. Pathophysiology of Bone Metastases

#### Tumor–Bone Microenvironment Interaction

a. Classification of bone metastases

**Figure 1:**
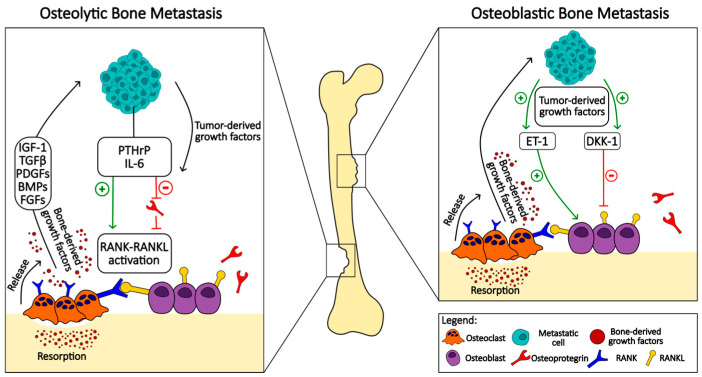
Comparative schematic representation of osteolytic and osteoblastic bone metastasis mechanisms: This figure illustrates the contrasting pathways and cellular interactions involved in osteolytic (**left panel**) and osteoblastic (**right panel**) bone metastasis. The osteolytic pathway is characterized by the destruction of bone tissue, primarily mediated by the activation of osteoclasts. Metastatic cells release tumor-derived growth factors, which stimulate the production of parathyroid hormone-related peptide (PTHrP) and interleukin-6 (IL-6). These factors promote the RANK-RANKL pathway, enhancing osteoclast maturation and activity, and leading to increased bone resorption. Bone-derived growth factors such as insulin-like growth factor 1 (IGF-1), transforming growth factor-beta (TGFβ), platelet-derived growth factors (PDGFs), bone morphogenetic proteins (BMPs), and fibroblast growth factors (FGFs) are released during bone matrix degradation, and they further stimulate tumor growth in a feedback loop. The osteoblastic pathway involves the formation of new bone tissue, which is typically dense and abnormal. Tumor-derived growth factors in this context stimulate osteoblasts directly and also induce the release of endothelin-1 (ET-1) and the inhibition of the Wnt signaling pathway antagonist, dickkopf-1 (DKK-1). This results in enhanced osteoblast activity and pathological bone formation. The figure also denotes the inhibitory effect of osteoprotegerin (OPG) on RANKL, which is not prominent in the osteoblastic pathway.

When cancer metastasizes to the bone, it leads to dysregulation of the physiological bone remodeling process [58]. Bone metastases can be broadly classified into osteoblastic or osteolytic lesions [59]. This classification is based on the radiographic appearance of bone metastases with osteoblastic lesions showing new bone formation and osteolytic lesions appearing with bone destruction. This represents two extremes of the effects on bone, with cases presenting with both lytic and blastic lesions (Figure 1). 

The main presentation of bone metastases in breast cancer is the radiologically identified process of bone tissue breakdown known as osteolytic lesions. These lesions result from the increased activity of osteoclasts that break down bone, leading to their weakening and becoming more subjective to pathological fractures. However, it is important to note that a significant minority, approximately 15 to 20 percent, of breast cancer metastases deviate from this usual pathway and instead induce the formation of new bone tissue. In this process, known as osteoblastic lesion, there is an abnormal proliferation of osteoblasts, the cells responsible for bone formation, leading to the development of sclerotic or dense areas in the bone [60]. In stark contrast, metastases from prostate cancer tend to primarily form osteoblastic lesions. This distinct pattern is characterized by the excessive formation of new bone tissue, albeit of structurally deviant and often functionally inadequate quality. Interestingly, despite the osteoblastic nature of these lesions, there is also evidence of increased bone resorption [60,61]. This contradictory situation is indicated by increased levels of bone turnover markers, suggesting an intricate interplay between the processes of bone formation and destruction associated with prostate cancer metastases.

Bone metastases associated with multiple myeloma also have a unique pathologic profile, as they are classified exclusively as osteolytic lesions. This exclusivity is attributed to a marked increase in osteoclast activity, which aggressively resorbs bone. This destructive process is further intensified by the simultaneous suppression of osteoblast function, which effectively obstructs the natural bone formation process and leads to the pronounced weakening of the bone structure. The pathological feature of multiple myeloma metastases emphasizes the critical imbalance between bone resorption and formation and highlights the aggressive nature of bone involvement in this malignancy [62,63].

The mechanisms of bone metastases are different in different types of cancer such as breast, prostate, and lung cancer, which illustrates the complexity and diversity of metastatic processes in these diseases.

Although both breast and lung cancers can lead to osteolytic lesions through increased osteoclast activity, the specific molecular mechanisms and the potential for osteogenic lesions in lung cancer differentiate the two. These differences emphasize the importance of tailoring therapeutic approaches to the specific cancer type and characteristics of bone metastasis. In breast cancer, estrogen receptor signaling pathways significantly influence the bone microenvironment and promote metastatic colonization and progression [64]. Lung cancer, particularly in women, may benefit from a bone microenvironment more favorable for metastasis. The overexpression of Notch3 is associated with bone metastasis in nonsmall cell lung cancer (NSCLC). This increases the expression and activity of ZEB-1, which facilitates epithelial–mesenchymal transition (EMT) and invasion [65]. 

A reduction in the ratio of androgens to estrogens has a particular effect on the bone metastases of prostate cancer, as it causes a “feminization” of the bone marrow, which promotes metastasis [66]. 

Prostate cancer metastases often exhibit more osteoblastic features, suggesting a unique interaction with bone tissue compared to other cancers. In addition, circulating tumor cells (CTCs) have been identified as predictors of bone metastasis in these cancers, highlighting their role in metastatic spread [3]. 

There are various targeted therapeutic approaches. In prostate cancer, the focus is on PIP5K1α inhibitors and GPRC5A, in breast cancer, on TGFβ and BMP signaling pathways, and in lung cancer, on the role of LIGHT in osteolytic destruction [67]. In addition, AKT activation by bone-derived factors is involved in all these cancers and promotes the ability to metastasize to bone through various signaling pathways and the secretion of bone cell-stimulating factors [68].

The different patterns of bone metastases seen in different types of cancer highlight the complex interplay between cancer cells and the bone microenvironment. This interplay not only influences the pathologic features of metastases but also has significant implications for the clinical management and therapeutic targeting of bone metastases in these malignancies. Understanding these mechanisms is crucial for the advancement of precise interventions aimed at alleviating the skeletal complications associated with cancer metastases, thereby enhancing patient outcomes and quality of life.

b. The metastatic cascade

The development of bone metastases can be described as a sequential, multistep process beginning with the tumor cells from the primary site locally invading the adjacent tissues (invasion) [69]. The tumor cells enter the bloodstream (intravasation). Once in circulation, these tumor cells, referred to as circulating tumor cells or CTCs, have the capacity to disseminate to various distant sites, including bone. Following their exit from the bloodstream (extravasation) and homing to premetastatic niches, these cells are now referred to as disseminated cancer cells or DCCs. DCCs settle in specialized microenvironments, or metastatic niches, namely the vascular and osteoblastic or endosteal niches, that facilitate their survival. Subsequently, DCCs either undergo apoptosis or survive and then undergo proliferation to form micrometastases or enter a dormant state [70]. Quiescent tumor cells escape from dormancy via several poorly understood mechanisms. DCCs proliferate and grow into micrometastatic bulk cells that are well adapted to the new bone microenvironment (colonization) [71]. Tumor-derived products lead to osteolysis, and the liberation of bone-derived factors that “feed” and support tumor cells that in turn will release further osteolytic factors, and the bidirectional interactions continue. This cycle is referred to as the vicious cycle of bone metastasis. Ultimately, the tumor cells grow into a macroscopic tumor and can alter the delicate balance of bone resorption and formation.

Evidence has shown that primary tumors actively induce the formation of a hospitable microenvironment, or premetastatic niches, remotely through the secretion of various factors [72,73]. Before the arrival of CTCs, primary tumors actively form premetastatic niches, which serve as predetermined destinations crucial for the “homing” of metastatic tumor cells in distant organs. Once settled in, CTCs interact with premetastatic niches, converting them into metastatic niches that are essential to the survival of metastatic tumor cells.

It is worth noting, however, that not all DCCs or micrometastases can grow to form an overt metastatic lesion owing to the inefficiency of the process of metastasis formation [74]. Only a few percent (around 0.02%) of disseminated tumor cells develop into clinically evident metastases [75,76]. The vast majority of DCCs fail at some step of the metastatic process due to the challenges and barriers faced in a harsh, new, and ever-changing microenvironment. In an analysis of bone marrow micrometastasis in breast cancer, the rate of DCCs detected in the bone marrow was higher than the proportion of patients who developed skeletal metastases [77]. Despite that, there is evidence suggesting that the presence of DCCs in the bone marrow predicts the development of skeletal metastases.

The state of dormancy affords DCCs their long-term survival and the evasion of immune surveillance through various mechanisms that continue to be explored [78]. Furthermore, dormancy confers resistance to conventional anticancer therapies that target rapidly proliferating cells [79]. Following treatment, dormancy enables the survival of tumor cells that persist as minimal residual disease (MRD), increasing the risk of recurrence. Years after the successful treatment of the primary tumor, relapses can occur due to cancer dormancy, which is particularly observed in patients with breast cancer metastases [80]. Similar observations have been made in prostate cancer metastases and various other cancers [81,82].

c. Bone is a preferred destination for metastases

Several factors have been found to play a role in the predilection of tumor cells to metastasize to bone [83]. The intrinsic properties of tumor cells and the characteristics of the bone microenvironment, as well as the interplay between them, increase the potential for skeletal metastases. This interplay, or crosstalk, between tumor cells and the bone microenvironment leads to the coevolution of both enabling osteolysis and metastasis growth. The mineralized extracellular matrix alongside the many cell types inhabiting the bone constitutes the bone microenvironment [84]. This environment represents a “fertile soil” in which disseminated cancer cells or “seeds” can survive and thrive [85].

The bone marrow is highly vascular and more accommodating to the bone colonization of circulating tumor cells [86]. The physical rigidity of the tumor microenvironment has been shown to be of importance to gene regulation and the transition of tumor cells to a bone-destructive phenotype. A rigid extracellular matrix increases the expression of the transcription factor GLI2, which in turn upregulates PTHrP expression through integrin β3 and TGFβ signaling in breast cancer cells, thus promoting cancer proliferation [87]. Physical force plays a role in supporting bone metastasis growth. Proliferating tumor cells exert pressure on the bone microenvironment. Osteocytes embedded within the bone matrix sense this pressure and upregulate chemokine ligand 5 (CCL5) and matrix metalloproteinases (MMPs), promoting the growth of prostate cancer bone metastases [88].

Moreover, a study explores how lower bone mineral density in breast cancer patients increases the risk of bone metastasis [89,90]. While the link between bone matrix mineralization and tumor cell behavior is not fully understood, mineralization-induced rigidity may drive metastatic progression. Surprisingly, experiments reveal that matrix mineralization prompts a less proliferative, stem-cell-like phenotype in breast cancer cells. In mouse models, bone mineral inhibits tumor growth and triggers a gene expression signature linked to extended metastasis-free survival. This suggests that changes in the bone matrix impact metastatic progression in breast cancer, emphasizing the need for in vitro models to integrate both organic and inorganic matrix components.

Bone is a reservoir of calcium and immobilized growth factors that are liberated by osteoclasts during bone resorption. TGFβ, IGF, FGF, BMP, VEGF, endothelin-1, and PDGF are among some of the growth factors released that aid metastatic tumor cells in their survival and growth [91,92]. Enhanced osteoclastic activity is then responsible for a vicious cycle in which the increased release of GFs leads to an increase in the frequency of metastasis [93]. The expression of adhesive molecules and receptor ligands on tumor cell surfaces allows for an increase in the frequency of bone metastases and homing. VCAM1 on tumor cells adheres to α4β1 on osteoclast progenitors, thus promoting osteoclastogenesis [94]. CXCR4, a protein found on tumor cells, binds to its receptor CXCL12, presents on bone marrow stromal cells, and induces tumor cell migration to bone [95,96,97].

## 3. Metabolic Reprogramming in the Microenvironment

### 3.1. Osteolytic Metastases

Osteoclasts, activated by tumor cell products rather than tumor cells, mediate the bone destruction seen in osteolytic lesions [98]. Different osteoclastogenic factors, both RANKL-dependent and RANKL-independent, have been implicated in different cancers [99]. The products of tumor cells activating osteoclastogenesis mostly achieve this via the upregulation of RANKL or the downregulation of OPG expression [100]. This tips the RANKL/OPG ratio favoring osteoclast-mediated bone resorption. In the intricate pathophysiology of osteolytic bone metastasis, the communication between osteoblasts and osteoclasts is mediated by membrane receptors Ephrin B2 and EphB4 [101]. These receptors, crucial for maintaining the equilibrium in bone remodeling, are disrupted in the presence of tumor cells, particularly in bone metastases. Ephrin B2, found on osteoclasts, and EphB4, found on osteoblasts and bone marrow stromal cells, play a pivotal role in regulating osteoclast activity and promoting osteoblast function. The altered interaction between these receptors contributes to the dysregulation observed in bone metastases.

The receptor activator of nuclear factor κB (RANK) and its ligand (RANKL) system are central players in osteoclast function. Tumor cells manipulate this system, predominantly through the secretion of parathyroid hormone-related protein (PTHrP) [102,103]. PTHrP, with a structure resembling parathyroid hormone (PTH), binds to its receptor (PTHR1) to increase RANKL expression. Multiple tumors show elevated PTHrP levels, including multiple myeloma, breast cancer, and prostate cancer [104,105,106]. PTHrP expression was greater at sites of bone metastasis compared to soft tissue metastases or the primary tumor site [107]. The binding of RANKL to RANK activates intracellular signaling pathways, including nuclear factor κΒ (NFκB), c-Jun, and melanogenesis-associated transcription factor, leading to osteoclastogenesis [101]. Despite the general consensus that PTHrP plays a promoting role in the growth and progression of bone metastases, some reports dispute this role. Both PTHrP and PTH act on the same receptor called PTH1R. In a study testing the effect of PTH on breast cancer bone metastases in a mouse model, it was reported that PTH treatment reduced tumor ingrowth into bone, reduced metastasis, preserved bone microarchitecture, and prolonged survival of mice [108]. This observation calls for caution and careful interpretation of the data, especially when considering the future use of these treatments in patient care. Osteoprotegerin (OPG), secreted by osteoblasts and bone marrow stromal cells, acts as a decoy receptor for RANKL, regulating its interaction with RANK. The balance between OPG and RANKL determines the extent of bone resorption [109].

Cytokines play a multifaceted role in osteolytic bone metastasis. Interleukins, such as IL-1, IL-6, IL-8, and IL-18, contribute to osteoclastogenesis and the promotion of osteolytic factors like PTHrP [101]. Tumor necrosis factor-α (TNFα), secreted by both tumor cells and bone marrow stromal cells, has a dual effect, promoting osteoclast differentiation while inhibiting osteoblast function. 

Canonical Wnt signaling plays a pivotal role in promoting bone metastasis, influenced by factors such as sclerostin and secreted frizzled-related protein 2 (SFRP2). Sclerostin, known for its inhibitory role in Wnt signaling, causes the disinhibition of the canonical Wnt signaling in metastatic breast cancer cells within the bone microenvironment [110]. Additionally, SFRP2, an upregulated glycoprotein in tumor vasculature, regulates Wnt signaling by binding to frizzled receptors on tumor endothelial cells, leading to angiogenesis through the noncanonical Wnt/Ca^2+^ pathway [111]. Targeting SFRP2 with antibodies shows antiangiogenic effects in tumor models, presenting a promising therapeutic avenue for inhibiting tumorigenesis and metastasis. Understanding these regulatory mechanisms provides insights into potential strategies for modulating canonical Wnt signaling in anticancer therapies. Moreover, DKK1, an inhibitor of the Wnt pathway, plays a dual role [101]. It inhibits osteoblast differentiation by suppressing the Wnt/β-catenin pathway and stimulates osteoclast activity by reducing osteoprotegerin (OPG) and enhancing RANKL expression [112]. In another recent publication, Wnt1 expression was shown to lead to osteoblastic changes in bone metastases in an established mouse model of osteolytic breast cancer. This observation thus provides direct evidence for the role of Wnt1 in this process, provides a model for further exploration of the molecular mechanisms underlying osteoblastic skeletal metastasis, and offers insights into potential therapeutic targets [113]. The dual and controversial action contributes to bone resorption and the progression of metastatic lesions. Targeting DKK1 and the Wnt pathway emerges as a potential therapeutic approach to counteract osteolytic bone metastasis.

The role of TGFβ in osteolytic bone metastasis is significant, particularly in the context of breast and prostate cancers [114]. TGFβ is present in the bone microenvironment, and metastatic cells release pro-metastatic cytokines that activate osteoclast differentiation. Activated osteoclasts degrade the bone matrix, releasing stored TGFβ. Notably, 75% of human bone metastasis biopsies exhibit active TGFβ signaling. In experimental assays, perturbations blocking TGFβ signaling, such as Smad4 knockdown or inhibitory Smad7 expression, dramatically reduce bone metastases in breast cancer and melanoma models. TGFβ promotes aggressive bone metastases by inducing pro-osteolytic factors like parathyroid hormone-related protein (PTHrP) [115]. TGFβ induces other genes like IL-11 and CTGF, part of the bone metastasis signature, and contributes to osteolytic metastasis. This creates a vicious cycle where TGFβ stimulates metastatic cells to activate osteoclasts, releasing more TGFβ and perpetuating bone metastatic lesions.

Jagged1 (JAG1) is another mediator of bone metastasis, particularly in breast cancer [116]. Functionally, JAG1 activates the Notch pathway in bone cells, fostering tumor growth by inducing IL-6 release from osteoblasts and promoting osteoclast differentiation. It operates downstream of the bone metastasis cytokine TGFβ, released during bone destruction. Treatment with a γ-secretase inhibitor disrupts the Notch pathway, diminishing JAG1-mediated bone metastasis. In various cancers, including breast cancer, high JAG1 expression correlates with advanced features and poor survival [117]. The JAG1–Notch pathway drives tumor progression through mechanisms like cyclin D1 regulation, the induction of epithelial–mesenchymal transition (EMT), and the enhancement of invasive ability. 

Osteocytes are the most abundant cell type in bone tissue. In recent years, it has been shown that the ability of osteocytes is to establish communication with cancer cells, hence exerting influence on their activity via an intricate network of signaling channels. This interaction promotes cancer cell survival, proliferation, and invasion, contributing to the progression of bone metastases [118,119]. In a multiple myeloma model, cancer cells upregulated the osteocyte production of fibroblast growth factor (FGF23) and vascular endothelial growth factor (VEGF-A) factors that are known to promote tumor proliferation and angiogenesis [120]. In the same myeloma model, osteocytes were shown to upregulate the expression of RANKL via Notch signaling, which, when inhibited, reduced osteocyte-induced RANKL upregulation [121]. A study was conducted to investigate the impact of mechanically stimulated osteocytes on the migratory and epithelial–mesenchymal transition (EMT) characteristics of breast cancer cells. However, the findings of this study have been conflicting [122]. In several reports, certain factors released by stimulated osteocytes, specifically CXCL1 and CXCL2 cytokines, have been found to enhance the migration of cancer cells [123]. However, contradictory findings have also been reported, suggesting that these osteocytes can actually reduce the expression of EMT genes and the ability of cancer cells for invasion and migration [124,125], thus highlighting the conflicting results in this area of research.

### 3.2. Osteoblastic Metastasis

Osteoblastic metastases, largely tied to prostate cancer but also observed in small-cell lung cancer, Hodgkin’s lymphoma, and medulloblastoma, represent a less-explored facet compared to osteolytic metastases [126]. In this type, bone mass increases due to heightened bone formation and reduced resorption, yet the functional integrity of the bone is compromised.

Tumor cells play a pivotal role in influencing the osteoblastic microenvironment. Platelet-derived growth factor (PDGF), consisting of A and B units, induces osteoblast differentiation and activity in prostate cancer bone metastases [101]. Fibroblast growth factors (FGFs) and vascular endothelial growth factors (VEGFs) further enhance osteoblast activity, contributing to altered bone homeostasis. The insulin-like growth factor (IGF I and II) exhibits increased levels in aggressive tumors, impacting osteoblasts. Notably, bone morphogenetic proteins (BMPs), especially BMPs 6, 7, and 4, secreted by tumor cells, stimulate both bone formation and angiogenesis.

The Wnt signaling pathway, modulated by Wnt ligands and the inhibitor dickkopf homolog-1 (DKK1), plays a crucial role in osteoblast differentiation [101,102]. Prostate cancer expresses Wnt 3a, 7b, 10b, and the Wnt inhibitor DKK1, maintaining a delicate balance between stimulatory and inhibitory signals within the Wnt pathway. Additionally, endothelin-1 (ET-1), a potent vasoconstrictor peptide secreted by tumors, stimulates abnormal bone formation through the endothelin A receptor (ETAR) and diminishes the production of the Wnt antagonist DKK1, activating the Wnt signaling pathway.

Urokinase plasminogen activator (uPA) and prostate-specific antigen (PSA) released by prostate cancer cells further contribute to osteoblastic activity [103,127]. uPA, in its high-molecular-weight form, enhances osteoblast activity by interacting with uPAR on osteoblasts. Moreover, PSA, a serine protease, modifies the NH2 terminal end of parathyroid hormone-related protein (PTHrP) and participates in the release of active forms of growth factors.

## 4. Altered Metabolism in Bone Metastasis

Metastasis, the leading cause of cancer mortality, depends on the adaptability of cancer cells to overcome obstacles in the microenvironment of distant organs [128]. The successful colonization of remote sites involves cancer cells navigating challenges like hypoxia, apoptosis, and immune clearance. Metastatic cells exhibit specific growth advantages influenced by genetic factors and microenvironmental conditions [129]. Metabolic reprogramming is a central feature of cancer cells as it enables the provision of the essential substrates and energy required for their survival and proliferation. This complex process involves the dysregulation of amino acid metabolism, a critical facet of cancer metabolism that is crucial for energy production, nucleoside synthesis, and the maintenance of redox homeostasis in cancer cells [130]. In addition, amino acid metabolism significantly contributes to the biochemical processes that are important for cancer cell growth and survival. Another notable aspect of this metabolic reprogramming is aerobic glycolysis, often referred to as the Warburg effect. This phenomenon is characterized by the fact that despite the presence of oxygen, glucose is predominantly converted to lactate via the glycolytic pathway rather than oxidative phosphorylation [131]. This shift in metabolic strategy not only underscores the flexibility and adaptability of cancer cell metabolism but also highlights the intricate interplay of different metabolic pathways in supporting the uncontrolled growth and proliferation of cancer cells, providing potential targets for therapeutic intervention. The metabolic reprogramming patterns vary among different metastatic cancers, reflecting the metabolic heterogeneity of tumor metastasis. Under various selective stresses, tumor cells with specific metabolic advantages selectively survive, leading to diverse metabolic phenotypes in different organs. In a study investigating the metabolic changes induced by the bone microenvironment in patient samples and prostate cancer cell lines, it was shown that PPP, and in particular its rate-limiting enzyme G6PD, are upregulated in the bone metastasis of prostate cancer, pointing to the specific role of the bone microenvironment in increasing the expression of G6PD via IL-6 secretion [132]. Cancer cell progression across various stages, including malignant transformation, tumor development, and metastasis, is influenced by mitochondrial reactive oxygen species (ROS). Mitochondrial ROS are implicated in tumor cellular adaptation to hypoxia, aggressive cell selection, and metabolic reprogramming to support cancer progression and colonization in distant organs [133,134]. 

### 4.1. Amino Acid Metabolism

Recent research highlights the connection between amino acid metabolism and bone metastasis, particularly in breast cancer [135]. The bone microenvironment offers a unique metabolic niche that influences cancer cells’ preferences for certain amino acids, impacting metastatic growth. Glutamine emerges as a key player, with bone metastatic breast cancer cells exhibiting a heightened dependence on glutamine compared to their primary counterparts [128]. Even in a high-glucose environment, these cells rely on glutamine for survival. The altered metabolic pattern in bone metastases involves the upregulation of enzymes like PHGDH, PSAT1, and PSPH, promoting glutamine utilization through serine biosynthesis. This shift is linked to the decreased expression of PKC-ζ [136]. Clinical relevance was established by linking the high expression of these genes to poor outcomes in breast cancer patients [137]. The study also highlighted the essential role of serine in cell proliferation and suggested its involvement in promoting osteoclastogenesis and bone destruction.

Despite being an essential amino acid, glutamine is abundant in the human body, particularly in the blood and muscles, constituting more than 20% of the free amino acid pool in the blood [138]. In the context of tumor cells converting glucose to lactic acid, glutamine plays a crucial role in supporting mitochondrial oxidative metabolism, sustaining energy metabolism, and inducing intracellular homeostasis. This high concentration of glutamine provides tumor cells with sufficient carbon and nitrogen sources, contributing to their uncontrolled growth. Additionally, glutamine contributes to various cellular activities, including amino acid synthesis, cell signal transduction, oxidative stress resistance, and immune escape. 

Glutamine contributes to the tricarboxylic acid (TCA) cycle and serves as a precursor in various biosynthetic pathways [139]. The dependence on exogenous glutamine is highlighted in specific cancer types, such as renal cell carcinomas (RCCs) and gliomas with IDH1 mutations, where glutamine-derived α-ketoglutarate (α-KG) sustains essential biosynthesis. Glutaminolysis, facilitated by glutaminase, becomes a vital process for cancer cells exhibiting glutamine addiction. The use of glutamine-based positron emission tomography (PET) tracers emphasizes the significance of glutamine as an alternative metabolic substrate for certain cancers. The concept of anaplerosis, where cells replenish TCA cycle intermediates, underscores glutamine’s role as a carbon and nitrogen donor in maintaining mitochondrial activity. Glutamine metabolism influences the synthesis of crucial molecules like proteins, lipids, and nucleic acids, and it plays a key role in mitigating oxidative stress through glutathione production. Inhibiting glutamine metabolism, specifically via glutaminase inhibition, has been explored as a therapeutic strategy to induce reactive oxygen species (ROS) overproduction, potentially leading to cancer cell devastation. Recent studies have uncovered alternative pathways for glutamate production in cancer, such as N-acetyl–aspartyl–glutamate (NAAG) hydrolysis and the glutaminase II pathway, providing new targets for potential combination therapies in cancer treatment.

Notably, studies have emphasized the significance of glutamine metabolism in tumor metastasis, where distant metastasis is a defining feature associated with advanced tumor staging and poor prognosis. Glutamine, which influences metastasis, facilitates energy provision for circulating tumor cell spread, protects against anoikis apoptosis, and shields cells from immune attacks in a colonization microenvironment.

Additionally, the enhanced catabolism of branched-chain amino acids (BCAAs), facilitated by BCAT1, is implicated in promoting bone metastasis by sustaining energy supply and activating stemness-related pathways [135]. BCAAs serve as both carbon and nitrogen sources for energy production, nucleotide synthesis, and protein synthesis [140]. Their metabolism is disrupted in multiple tumors, often associated with alterations in enzymes, transporters, and transcription factors involved in the BCAA metabolic pathway. BCAT1, a key player in BCAA metabolism, is frequently altered in tumors and has become a target for diagnostic and therapeutic interventions.

Kynurenine (KYN) is a metabolite of the amino acid l-tryptophan. It influences osteoclastogenesis through the activation of the aryl hydrocarbon receptor (AhR) pathway [141]. In Raw 264.7 cells, a murine macrophage line, KYN enhances RANKL-mediated osteoclast differentiation. KYN treatment increases the number of multinucleated TRAP+ osteoclasts and bone resorption activity. This effect is mediated by the upregulation of c-fos and NFATc1, key transcription factors in osteoclast differentiation. By blocking AhR signaling, it reduced KYN/RANKL-induced osteoclast formation, underscoring the crucial role of AhR in this process [142]. This observation suggests potential therapeutic targets for bone diseases such as bone metastases by modulating KYN and AhR pathway activities, which could be another way to stop or delay bone metastases.

### 4.2. Glucose Metabolism

In the intricate landscape of bone metastasis, cancer cells orchestrate specific metabolic adaptations, prominently centered around glucose metabolism, to fuel their growth and resilience within the bone microenvironment [143]. Glucose, primarily transported by glucose transporters 1 and 3, emerges as a pivotal bioenergetic substrate essential for supporting bone resorption—a fundamental process in metastatic development within bones. Notably, the dose-dependent effects of glucose on osteoclastogenesis are intricate, with optimal levels stimulating the formation and resorptive activity of osteoclasts. However, at elevated concentrations, glucose exerts inhibitory effects on these processes. One striking metabolic alteration observed in bone metastasis is the Warburg effect, characterized by cancer cells favoring aerobic glycolysis even in the presence of oxygen [144]. This metabolic reprogramming leads to an upsurge in glycolytic activity, culminating in heightened lactate production and increased glucose consumption. Initially considered a secondary effect to genetic and epigenetic alterations, it is now recognized that altered cellular metabolism fuels tumor growth by providing substrates for biosynthesis. Cancer cells prioritize glycolytic intermediates for anabolic pathways, supporting continuous cell division. Oncogenes and tumor suppressors not only drive cell proliferation but also coordinate cellular metabolism to meet biosynthetic demands [145]. Anoikis is a form of programmed cell death triggered by the loss of cell–matrix interactions. In the context of metastasis, the Warburg Effect equips cancer cells with enhanced resistance to anoikis. Detached cancer cells, characterized by increased glycolysis and reduced glucose oxidation, efficiently regulate reactive oxygen species (ROS) levels, mitigating oxidative stress-induced anoikis. This metabolic adaptation ensures that cancer cells can withstand the challenges of matrix detachment, facilitating their survival during metastatic progression. In essence, the Warburg Effect not only fuels uncontrolled cell proliferation but also confers a vital resistance mechanism to anoikis, promoting the metastatic potential of cancer cells. 

The hypoxic and acidic microenvironment of bone metastasis further accentuates this glycolytic shift. This reliance on aerobic glycolysis not only fulfills the energy demands of metastatic cells but also results in increased lactate secretion, contributing to extracellular acidification. The intricate interplay between cancer cells and the bone microenvironment, marked by heightened glycolysis and lactate production, creates an environment conducive to enhanced bone resorption and metastatic progression. 

The Warburg effect assumes a central role in the evolution of cancer cells, influencing the cellular energy metabolism and actively participating in the modulation of key transcriptional factors and proteins like FOXM1, p53, NFκB, HIF1α, and c-Myc [146]. Notably, glycolytic enzymes, including GLUTs, HKs, PFKs, LDHs, and PKM2, make substantial contributions to the development of cancer, with their elevated expression being associated with advanced tumor stages and the occurrence of metastasis. Additionally, noncoding RNAs, such as lncRNAs, miRNAs, and circular RNAs, play pivotal roles in overseeing the glucose metabolism of cancer cells, fostering processes like growth, proliferation, and metastasis.

Osteoclast differentiation involves increased glucose and oxygen consumption, the upregulation of metabolic enzymes, and mitochondrial biogenesis stimulated by PGC-1β [143]. During resorption, glucose transport and ATP levels rise significantly, with AMPK and mTOR playing crucial roles in osteoclast differentiation and function. Changes in nutrient availability during osteoclast differentiation can notably influence AMPK, mTORC1, and mTORC2 complexes, suggesting that alterations in metabolic substrates due to the presence of cancer cells may directly impact osteoclast behavior.

## 5. Clinical Features of Bone Metastases

### 5.1. Bone Pain

Cancer-induced bone pain (CIBP) affects 60–84% of advanced cancer patients because of inflammatory and mechanical factors [147,148]. Inflammatory pain results from cytokine release and nerve stimulation, while mechanical pain stems from tumor mass pressure, causing bone weakness and activity-related pain [148]. Pain severity rarely aligns with lesion extent, and breakthrough pain, triggered by movement, adds complexity [147,149]. Mechanisms involve tumor, bone matrix, and inflammatory cell interactions, activating nociceptors, damaging nerves, and inducing growth factors [147]. Osteoclasts and tumor cells contribute to bone destruction, and neurotrophins, cytokines (IL-1β and TNFα), and other factors (ATP, TGFβ1, IGF-1, and sclerostin) play roles, suggesting potential intervention targets for improved pain management and enhanced patient quality of life [150].

### 5.2. Hypercalcemia

Hypercalcemia in malignancy affects up to 44.1% of cancer patients. It is primarily driven by parathyroid hormone-related protein (PTHrP) or the receptor activator of nuclear factor kB ligand, with bone metastases and increased osteoclastic activity contributing to 20% of cases [151,152]. Despite often presenting mild symptoms, hypercalcemia can lead to rapid metabolic derangements and significant morbidity, causing approximately 50% of affected cancer patients to pass away within 30 days [153]. Osteoclasts, not tumor cells, play a central role in bone destruction in osteolytic metastases, as tumors stimulate local osteoclast production and activity [154]. Symptomatic hypercalcemia can manifest in neuropsychiatric, gastrointestinal, renal, and cardiovascular systems, contributing to pain experienced by cancer patients [155].

### 5.3. Pathological Fractures and Spinal Compression

Pathologic fractures, affecting 10–30% of cancer patients, predominantly involve the femur in over 50% of cases, with breast cancer causing 60% and lung cancer 10% [148]. In individuals with known bone metastases, the sudden onset of pain prompts swift assessment for potential fractures, even without deformity [156]. Predictors include large lytic lesions, with Mirels’ scores ≥10 indicating a fracture risk over 50% and likely surgery [151]. Back pain has been described to be due to spinal instability in 10% of the cases [157]. A Spinal Instability Neoplastic Score has been established to define the cases where surgical intervention might be beneficial [158,159]. Spinal cord compression occurs in 5–10% of cancer patients and up to 40% of those with nonspinal bone metastasis, leading to neuropathic pain [149,160]. Metastasis to the vertebra, common due to a rich blood supply, can cause malignant spinal cord compression (MSCC), an oncologic emergency, resulting in outcomes from pain to paraplegia [157]. MSCC diagnosis is often delayed, taking approximately 2 months from pain onset. 

## 6. Early Detection

Bone metastases in advanced solid tumors lead to debilitating skeletal events, profoundly impacting survival and the quality of life [161]. On average, untreated bone metastases result in such events every 3–6 months, emphasizing the need for timely detection to shift focus from palliation to prevention. Challenges, including the absence of screening programs, hinder early identification in advanced cancer patients. Examining three presentation scenarios revealed that patients with an identified primary malignancy at skeletal metastasis diagnosis had the most prolonged survival (14 months), while those with an unknown primary had a less favorable prognosis [162]. The early detection of bone metastasis is crucial for improved survival rates, especially as metastases of unknown origin occur in 3 to 4% of all malignancies.

## 7. Challenges

Despite the proven benefits of early detection, numerous challenges hinder the timely diagnosis of bone metastasis. Up to 50% of early cases show no symptoms, allowing silent progression unless primary tumor identification occurs [149]. Plain radiographs, often used for the initial evaluation of patients with bone pain, are insensitive to screening asymptomatic metastases [163]. Common clinical features with other bone diseases make bone pain an unspecific symptom. Radiological evaluations, including plain X-rays and CT scans, face challenges in sensitivity and specificity, with mimickers like multiple myeloma, complicating interpretation [149]. Bone scans, though valuable in detecting osteoblastic activity, may yield false negatives in lytic metastases. MRI and PET scans offer greater sensitivity and specificity but are not routinely performed without specific indications. 

## 8. Diagnosis

### 8.1. Imaging

Several imaging techniques are currently available to diagnose bone metastases. Radiographs, while useful for screening and predicting fracture risk, have limited sensitivity (44 to 50%) and may not conclusively rule out bone metastasis with a high pretest probability [148]. Lytic metastases show a permeative lesion, while osteoblastic lesions exhibit a sclerotic appearance and visible fracture lines [164]. Radiographs, less sensitive than bone scans or FDG-PET scans, need significant bone density reduction for lesion visibility [163]. Survey radiography can detect 90% of skeletal metastases from breast cancer in the skull, chest, spine, and pelvis [165].

Computed tomography (CT) scans, with a sensitivity of 74% and specificity of 54%, are crucial for diagnosing bone metastasis, especially when there is a suspected fracture or spinal cord compression [158,163]. CT provides detailed bone information for treatment planning, detecting bone metastases before radiographic evidence, and assessing fracture risk [159]. It has a similar sensitivity to bone scans but offers high-resolution views of the bone cortex [166]. 

Magnetic resonance imaging (MRI) is crucial for diagnosing bone metastasis, offering heightened sensitivity compared to CT. With a per-lesion sensitivity and specificity of 91 and 96 percent, MRI outperforms CT and bone scans, excelling remarkably in spinal metastasis assessment [159]. MRI is valuable for assessing spinal cord compression, nerve root impingement, and discerning benign and malignant compression fractures [167]. MRI’s detailed information on bone lesions, soft tissues, and solid organs makes it invaluable, although it may not fully replace bone scans in certain scenarios.

Positron emission tomography (PET) scanning, specifically with FDG-PET and integrated FDG-PET/CT, is a highly valuable diagnostic tool for detecting distant bone metastasis from various solid tumors and multiple myeloma [168]. The metabolic imaging provided by FDG-PET, either alone or integrated with CT, shows notable sensitivity and specificity in clinical staging and restaging evaluations for metastatic diseases [169]. Integrated FDG-PET/CT improves sensitivity, as seen in breast cancer studies, enhancing per-lesion sensitivity and specificity rates compared to bone scans. 

Bone scan using Tc-99m skeletal scintigraphy, or “bone scan”, utilizes 99mTc-MDP to highlight areas of increased osteoblastic activity, providing a comprehensive skeleton examination with sensitivity ranging from 79% to 86% and specificity between 81% and 88% [170]. Limitations include reduced sensitivity for tumors with minimal osteoblastic activity, like multiple myeloma, and the potential oversight of lytic lesions [163]. 

### 8.2. Other Diagnostic Tools

The diagnosis of bone metastasis involves assessing elevated serum alkaline phosphatase, frequently seen in prostate and breast cancer cases [149,165,171]. Calcium and alkaline phosphatase concentrations indicate increased osteoblastic activity, aiding in cancer detection and treatment monitoring. Tumor markers, including PSA and others, play a crucial role in identifying the cancer type. Laboratory tests help rule out alternative diagnoses and assess complications like hypercalcemia [166]. Anemia and thrombocytopenia are common in metastatic bone disease, with PSA testing serving as an exception in older men with blastic lesions, indicating the prostate as the primary site. While serum alkaline phosphatase serves as a biomarker, other biomarkers like Fab isoenzyme and TRAcP b5 are considered, with limitations in specificity due to various factors [171]. 

Bone biopsies are the gold standard for diagnosing bone metastasis definitively [171]. In cases with a known primary tumor and typical skeletal lesions on imaging, a biopsy may not be mandatory but may carry prognostic implications and the possibility of benign or second occult primary lesions [172]. Needle biopsy, especially CT-guided fine-needle aspiration biopsy (FNA), is valuable for confirming metastatic disease in patients with a history of cancer but no previous bone metastases [173]. Core biopsy, although providing higher diagnostic accuracy for tumor type and grade, may not be essential in some cases. Biopsies are indicated for patients with an unknown primary cancer presenting with bone metastasis to confirm malignancy and identify the primary site [149,165,166]. Liquid biopsies based on genetic analysis offer a minimally invasive alternative [173]. 

## 9. Management

Existing therapeutic approaches for bone metastases aim to induce tumor regression, impede tumor cell proliferation, or mitigate the impact of cancer cells in the bone, which can cause bone degradation and associated complications like fractures, bone pain, spinal cord compression, and hypercalcemia. The term “skeletal-related events” refers to a collection of symptoms, as previously mentioned. Existing treatments for bone metastasis are not therapeutic and mostly focus on providing palliative care [174]. This section will cover several therapeutic approaches for treating individuals with bone metastases. 

### 9.1. Bone Pain Management

The management of bone pain in bone metastasis involves a three-step approach following the World Health Organization’s analgesic ladder, starting with nonopioids and progressing to strong opioids [149,175]. NSAIDs, alone or combined with opioids, are effective for short-term cancer pain. Neuropathic pain may respond to anticonvulsants or tricyclic antidepressants. Opiates are the mainstay for pain, and their appropriate titration is essential. NSAIDs are used to address mild or breakthrough pain, and short courses of steroids are reserved for nerve irritation [166]. There is limited evidence for weak opioids, while recommendations suggest using strong opioids such as buprenorphine, oxycodone, fentanyl, morphine, and methadone for severe pain. 

### 9.2. Bone Resorption Modulators

Bisphosphonates, potent inhibitors of osteoclast-mediated bone resorption, bolster bone stability in the context of bone metastases, drastically reducing fracture rates, the need for radiotherapy, and hypercalcemia [171]. However, they have limited impact on spinal cord compression rates and are associated with side effects. Denosumab, a RANKL inhibitor, prevents osteoclast formation and activation, exhibiting advantages over bisphosphonates in reducing skeletal-related events and pain [175,176,177]. Medical professionals consider both treatments as essential in the management of bone metastasis. Various systemic approaches, including mammalian target of rapamycin inhibitors and proteasome inhibitors, modulate osteoclastogenesis, contributing to overall bone metastasis care [177]. While newer modalities show promise, the focus is on bisphosphonates and denosumab as standard care for preventing skeletal-related events and modulating osteoclast activity in bone metastasis.

### 9.3. Hormone Therapy

Hormone therapy can play a pivotal role in managing bone metastasis, especially in breast and prostate cancer [166]. Strategies like bilateral oophorectomy, LH-RH antagonists, and antiestrogens such as tamoxifen have demonstrated success in breast cancer, yielding regression rates of 17 to 35 percent in sensitive tumors. For metastatic prostate cancer, initial treatments involving diethylstilbestrol or LH-RH agonists have proven effective in reducing pain and improving ambulatory status in 40 to 70 percent of patients. 

### 9.4. Radiation Therapy

Radiation therapy effectively manages bone metastasis, providing palliative relief and promoting bone healing [149,165]. The use of palliative radiotherapy is warranted when patients with bone metastases experience bone pain that does not effectively respond to analgesia. While caution is advised to avoid compromising fracture healing, pain relief is notable, especially in breast, prostate, or pulmonary neoplasm cases [165]. 

### 9.5. Radiopharmaceuticals 

Radiopharmaceuticals, including strontium-89, samarium-153, rhenium-186, radium-223, and 177Lu-PSMA-617, play a role in managing metastatic bone pain [166,171,178]. These agents selectively deliver radiation to areas with increased bone activity, targeting multiple metastases. Radium-223 stands out for its alpha-emitting properties, improving survival and controlling bone pain [178]. The use of radionuclides becomes essential when bone metastases are widespread [149]. Combining radionuclides with bisphosphonates or denosumab and innovative compounds like [68Ga/177Lu]DOTAZOL enhances possibilities in bone-targeted imaging and therapy [178].

### 9.6. Radiofrequency Ablation

Radiofrequency ablation (RFA) offers a minimally invasive and effective approach for relieving pain in bone metastasis (BM), with a success rate of around 95% [175]. By using high-frequency alternating current, RFA targets abnormal tissue, reducing pain through mechanisms like inhibiting pain transmission and shrinking tumor volume [149]. It is particularly useful for refractory pain in osteolytic BM not responding to standard treatments [171]. Although potential risks include nerve and spinal cord damage, RFA is generally considered safe and reliable, providing rapid pain relief with documented complications ranging from 0 to 6.9% [171,179]. 

### 9.7. Chemotherapy

Chemotherapy is another player in addressing bone metastasis (BM) across various tumors like breast cancer and lymphoma [171]. Its effectiveness depends on factors such as histology and chemosensitivity. Electrochemotherapy, combining electric pulses and drugs, provides pain relief for resistant BM cases [176]. Neoadjuvant chemotherapy aims to reduce tumor size and pulmonary metastases. In systemic treatment, chemotherapy complements radiotherapy, with evolving approaches like stereotactic body radiation holding promise for efficient and safe BM management [180].

### 9.8. Surgery

Surgery is another intervention for addressing bone metastasis (BM), primarily aiming to relieve pain, improve function, and occasionally extend survival [166,181,182]. Indications for surgery include neurological deficits, spinal instability, unmanageable pain from resistant tumors, and unstable fractures [183]. The main surgical goal is nerve decompression, spine stabilization, and reconstruction, with various approaches employed based on tumor characteristics [176]. Minimally invasive techniques such as radiofrequency ablation and percutaneous vertebroplasty are employed by surgeons for vertebral collapse. Surgical options range from simple decompression to en bloc resection and fixation, tailored to the region involved and patient factors [176]. 

### 9.9. Targeted Therapies

**Figure 2:**
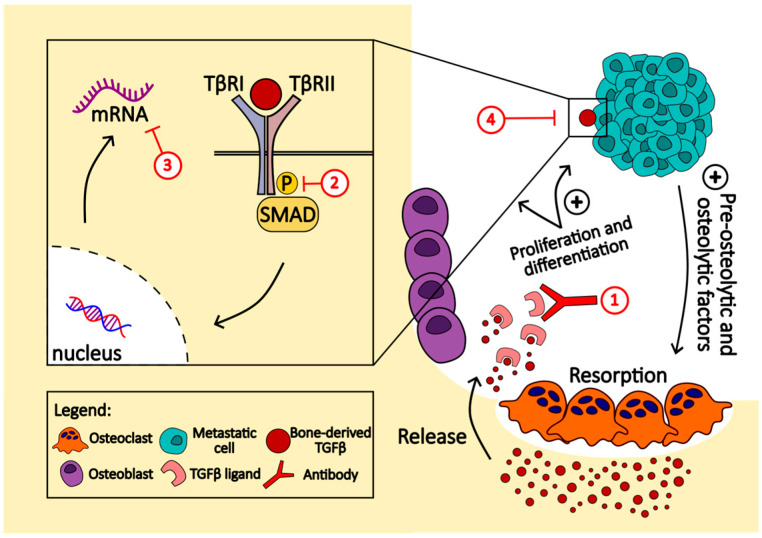
This figure highlights the vicious cycle of bone metastasis driven by TGFβ signaling, emphasizing the potential for therapeutic intervention in this pathway to disrupt the cycle of bone destruction and tumor growth promotion. Several approaches can be used to target this pathway. (1) TGFβ ligands are neutralized or trapped using soluble decoy receptors or neutralizing antibodies; (2) TGFβ receptor kinase inhibitors, like SD-208 and LY2109761, act via ATP-competitive inhibition; (3) using antisense oligonucleotides (ASO), such as Ap-12009 (trabedersen), offers another strategy to reduce TGFβ levels by inhibiting mRNA function and protein synthesis; (4) biologic-based molecules like BMP7 counteract TGFβ-induced epithelial–mesenchymal transition and inhibit the formation of bone metastases.

Targeted therapies for bone metastases involve various critical signaling pathways. The transforming growth factor-beta (TGFβ) family and Smad signaling play crucial roles in both cancer progression and bone homeostasis [184]. Targeting TGFβ is hypothesized to have dual antitumor and bone-protective effects. One approach is to physically neutralize or trap TGFβ ligands using soluble decoy receptors or neutralizing antibodies. Monoclonal antibodies demonstrate therapeutic potential by binding and reducing the biological activity of TGFβ isoforms. Antisense oligonucleotides (ASOs) offer another strategy to reduce TGFβ levels by inhibiting mRNA function and protein synthesis. TGFβ receptor kinase inhibitors act via ATP-competitive inhibition and have shown promise in preclinical bone metastasis models (Figure 2) [185]. Additionally, biologic-based molecules like BMP7 counteract TGFβ-induced epithelial–mesenchymal transition and inhibit the formation of bone metastases. Halofuginone (Hfg), known to inhibit TGFβ signaling, has shown efficacy in inhibiting osteolytic lesions and bone metastases in preclinical models [186].

The bone morphogenic protein (BMP) pathway, integral to mesenchymal stem cell differentiation, shows mixed roles in cancer, with pharmacological inhibition showing promise in certain bone metastasis models [187,188]. The Wnt signaling pathway, crucial in osteogenesis and cancer metastasis, offers potential therapeutic targets, including various inhibitors that are being investigated for their efficacy in prostate and breast cancers [189]. The CXCL12/CXCR4 axis, significant in bone metastasis regulation, has inhibitors in clinical trials showing potential to reduce cancer cell proliferation and migration [190,191]. Lastly, the PI3K/AKT pathway, vital for osteoblast and osteoclast survival, demonstrates potential in bone metastasis management, with inhibitors like alpelisib approved for specific cancer mutations [192]. This section emphasizes the ongoing research and development of targeted therapies for more effective management of bone metastases.

## 10. Future Directions in Bone Metastasis Research and Treatment

### 10.1. Novel Diagnostic Techniques

#### 10.1.1. Liquid Biopsy

Liquid biopsy represents a transformative advance in diagnosing bone metastasis, offering a minimally invasive method to detect circulating tumor DNA (ctDNA), circulating tumor cells (CTCs), and extracellular vesicles (EVs) [193,194]. This technique has demonstrated potential in monitoring metastatic progression in breast, prostate, and lung cancers by providing a genetic snapshot of the tumor’s mutational landscape, which correlates with patient outcomes and response to treatment. For instance, ctDNA analysis can anticipate clinical recurrence almost a year in advance in breast cancer patients, with levels directly proportional to survival rates. Similarly, CTC counts have prognostic significance in lung cancer, indicating the presence and extent of bone metastasis. EVs, enriched with specific miRNAs or mRNAs, have emerged as predictive tools for early detection in both breast and lung cancers. Despite its promising utility, the application of liquid biopsy in clinical practice faces challenges, including sensitivity, specificity, and the need for standardized protocols [194]. As research progresses, refining liquid biopsy techniques could greatly enhance early diagnosis, treatment monitoring, and personalized therapy for bone metastasis, marking a remarkable step forward in oncological care.

#### 10.1.2. Advanced Imaging Modalities

Integrating artificial intelligence (AI) in imaging for the diagnosis of bone metastasis signifies a revolutionary leap forward, harnessing deep learning algorithms to enhance sensitivity and specificity in identifying osseous metastases across various imaging modalities like WBS, CT, MRI, and PET/CT [195]. AI’s ability to analyze medical images, detect subtle changes in bone tissue texture, and segment lesions advances the precision of diagnosis and treatment planning. Despite the promising outlook, AI’s application in bone metastasis detection confronts challenges, such as the need for extensive and diverse datasets for algorithm training, potential bias, overfitting, and the quest for standardized evaluation metrics to ensure the reliability and accuracy of AI models. The call for interpretable machine learning further stresses the demand for transparent, understandable AI processes, especially in clinical settings where decision making is critical. The pursuit of high-performance medicine through AI aims to amalgamate representation learning with complex reasoning, propelling beyond the current capabilities of human intelligence to a future where diagnosis and treatment of bone metastasis are significantly data-driven and efficient.

#### 10.1.3. Metabolic Imaging

Hyperpolarized 13C-pyruvate MRI (HP 13C-MRI) is revolutionizing the field of metabolic imaging in bone metastasis diagnosis by allowing for the real-time observation of metabolic activities within tumors, offering insights far beyond the capabilities of conventional imaging methods [196,197]. This novel technique capitalizes on the Warburg effect, highlighting tumor aggressiveness and therapeutic responses through the measurement of pyruvate-to-lactate conversion rates. Its ability to correlate with tumor biopsies and complement traditional imaging findings underscores its potential to enhance treatment strategies and patient outcomes. Despite its promise, the need for specific infrastructure and the necessity of validation through extensive research challenges its widespread adoption. As this technology matures, it is anticipated to become integral to diagnostic processes, improving the detection of metastases and the tailoring of oncologic treatments [197]. The integration of HP 13C-MRI into clinical practice holds the promise of transforming the landscape of cancer diagnosis and management by providing a noninvasive, real-time window into tumor metabolism.

#### 10.1.4. Emerging Therapies

##### Immunotherapy

Immunotherapy presents a promising yet challenging approach for treating bone metastases, hindered by the bone marrow’s immunosuppressive environment, which protects against autoimmune insults but also limits immunotherapy’s effectiveness [198]. Strategies to overcome these challenges include targeting the immunosuppressive mechanisms, such as the role of mesenchymal stem cells (MSCs) and the TGFβ pathway, which inhibit antitumor immune responses. Innovations in cell-based therapies, like CAR-T and TCR-T cell technologies, aim to improve the targeting of the bone microenvironment, enhancing therapy efficacy. Clinical observations suggest that combining immune checkpoint blockade (ICB) with bone-targeted therapies or modifying the gut microbiota could potentiate the antitumor activity of cytotoxic T cells. The dynamic expression of PD-L1 and the presence of primary and acquired resistance to ICBs call for the identification of new biomarkers and the development of comprehensive assessment criteria for bone metastases [199]. The combination of ICIs with bone-targeted therapies (BTTs), chemotherapy, antiangiogenetic drugs, or bisphosphonates/denosumab could offer therapeutic advantages by transforming the “cold” tumor microenvironment to a “hot” one, potentially overcoming resistance to immunotherapy [200]. Despite initial successes, further research is necessary to optimize these approaches, overcome resistance, and identify reliable biomarkers for predicting treatment outcomes.

##### Targeted Molecular Therapies

Advances in the genomic and proteomic profiling of bone metastases have identified the key pathways driving osteolytic and osteoblastic processes. Novel agents targeting specific molecular drivers, such as Src kinase inhibitors, Wnt pathway inhibitors, and RANKL pathway modulators, are under investigation [201,202,203]. Exploring the RANKL/RANK pathway reveals its potential to revolutionize treatment across various cancers, including breast cancer, melanoma, and NSCLC, especially when combined with immune checkpoint inhibitors and other targeted therapies [202]. This approach could enhance treatment efficacy and overcome resistance in tumors less responsive to current immunotherapies. Concurrently, Src inhibitors like saracatinib and dasatinib are being evaluated for their ability to mitigate skeletal-related events and directly inhibit tumor growth in metastatic bone disease, promising a new direction in combination therapy strategies. These therapies aim to disrupt the vicious cycle between tumor cells and bone remodeling cells, offering a targeted approach to impede metastatic progression.

##### Bone-Targeting Radiopharmaceuticals

Bone-targeting radiopharmaceuticals (RPs) are emerging as a promising therapy for bone metastasis, specifically in prostate cancer, offering pain palliation and potential survival benefits [204]. The safety and efficacy of α-emitter Ra-223 have been confirmed for castration-resistant prostate cancer (CRPC) with bone metastasis, which prompts the need for further trials to explore its utility in other cancers. Current research focuses on combining radionuclide therapy with chemotherapies or immune checkpoint inhibitors (ICIs) to enhance patient survival, a strategy supported by ongoing phase III trials [205]. However, combining radium-223 with certain treatments has revealed challenges, such as treatment-emergent fractures and increased mortality, underscoring the need for the cautious evaluation of combination therapies. Additionally, the feasibility of retreatment with RPs and their integration with standard chemotherapy regimens are subjects of ongoing investigation. These efforts aim to establish more effective, safe treatment protocols that not only alleviate bone pain but also extend survival for patients with metastatic bone disease.

##### Perspective Analysis

In addressing the critical aspects of treatment modalities for bone metastasis, it is imperative to delve into the nuanced roles of anticancer therapies and the pivotal influence of the immune response. The landscape of bone metastasis management is complex, with treatments ranging from chemotherapy and hormone therapy to targeted therapies and immunotherapies. Each modality offers unique mechanisms of action against tumor cells within the bone microenvironment, necessitating a detailed exploration of their efficacy, challenges, and interplay with the host’s immune system.

Anticancer therapies target tumor regression and mitigate the proliferation of cancer cells. However, their role extends beyond these objectives, particularly in the context of bone metastasis. For instance, the direct effects of chemotherapy and hormone therapy on the bone microenvironment are critical yet underexplored. These treatments can inadvertently influence bone remodeling processes, potentially altering the progression of metastases. Moreover, the advent of targeted therapies has introduced a precision medicine approach, focusing on specific molecular pathways implicated in bone metastasis. Yet, the critical analysis of their long-term outcomes, potential resistance mechanisms, and effects on bone health remains scant.

Integrating immunotherapy into the treatment regimen for bone metastasis marks a significant paradigm shift. The immune system’s dual role in tumor promotion and suppression within the bone microenvironment warrants a nuanced understanding. Immunotherapies, such as checkpoint inhibitors, aim to unleash the immune system against cancer cells. However, the efficacy of these treatments in the context of bone metastasis is contingent upon a delicate balance. The potential for these therapies to remodel the immune landscape in favor of tumor suppression is promising, yet challenges such as immune-related adverse events and the complexity of immune evasion mechanisms by tumor cells necessitate a critical evaluation.

Recent clinical trials and preclinical studies have shed light on the efficacy of novel treatment modalities in managing bone metastasis. These studies provide a wealth of data on patient outcomes, including survival rates and the quality of life. However, a critical analysis reveals gaps in our understanding of the long-term effects of these treatments on the bone microenvironment and immune response. The promise shown by combination therapies, for instance, underscores the potential for synergistic effects, yet the optimal combinations and sequencing of treatments remain areas of active investigation.

The exploration of the immune response’s relevance in bone metastasis treatment unveils a complex interplay of immune cells, cancer cells, and bone tissue. Emerging research underscores immunotherapy’s potential to enhance the immune system’s capacity to target bone metastases. Nonetheless, the critical analysis of these therapies must consider the heterogeneity of the immune landscape across different patients and tumor types. The dynamic interactions between cancer cells and the immune system in the bone microenvironment, particularly the mechanisms of immune evasion and the potential for immunosuppression by certain therapies, highlight the need for a tailored approach to treatment.

The management of bone metastasis is at a pivotal juncture, with anticancer therapies and immunotherapies offering new avenues for treatment. However, a critical analysis underscores the need for a deeper understanding of these treatments’ effects on the bone microenvironment and immune response. Future research directions should focus on elucidating the mechanisms of action of these therapies, optimizing treatment combinations, and addressing the challenges posed by immune evasion strategies. By bridging these gaps, we can move toward a more effective and nuanced approach to the management of bone metastasis, ultimately improving patient outcomes.

## 11. Conclusions

In this article, we delved into the intricate relationship between cancer cells and the bone microenvironment, revealing how this interaction leads to bone metastases. This process is complex, involving both the breakdown (osteolytic) and building (osteoblastic) of bone tissue, each pathway bringing its own unique challenges and effects. This review highlights the significant role that systemic factors like hormones and cytokines play in bone health and remodeling. By gaining a deeper understanding of bone metastases, including their clinical characteristics, diagnostic challenges, and treatment options, we greatly enhance our knowledge of this complex condition. This not only improves our grasp of bone metastasis but also opens the door to new, innovative treatment methods. These methods aim to improve patient outcomes by simultaneously targeting cancer cells and the altered bone environment.

Looking ahead, further research is needed to unravel the detailed molecular mechanisms of bone metastases, particularly focusing on how tumors interact with the bone microenvironment. Identifying key biochemical pathways and players will help in developing targeted therapies that could more effectively treat bone metastases. Additionally, a better understanding of metabolic changes within the tumor environment may reveal new ways to slow down the progression of bone metastases. The ultimate goal is to enhance the quality of life and increase survival rates for patients by creating better diagnostic tools and treatments that address both metastatic cancer and associated bone issues. Thus, this field remains a promising and vital area of research, offering the potential to significantly improve the management and prognosis of cancer patients with bone metastases.

## Data Availability

Not applicable.
